# High relatedness of invasive multi-drug resistant non-typhoidal *Salmonella* genotypes among patients and asymptomatic carriers in endemic informal settlements in Kenya

**DOI:** 10.1371/journal.pntd.0008440

**Published:** 2020-08-03

**Authors:** Samuel Kariuki, Cecilia Mbae, Sandra Van Puyvelde, Robert Onsare, Susan Kavai, Celestine Wairimu, Ronald Ngetich, John Clemens, Gordon Dougan

**Affiliations:** 1 Centre for Microbiology Research, Kenya Medical Research Institute, Nairobi, Kenya; 2 Department of Biomedical Sciences, Institute of Tropical Medicine, Antwerp, Belgium; 3 Laboratory of Medical Microbiology, Vaccine & Infectious Disease Institute, University of Antwerp, Antwerp, Belgium; 4 Parasites and Microbes, Wellcome Sanger Institute, Wellcome Genome Campus, Hinxton, Cambridge, United Kingdom; 5 Office of the Executive Director, International Diarrheal Diseases Research Centre, Dhaka, Bangladesh; 6 Cambridge Institute for Therapeutic Immunology & Infectious Disease, Department of Medicine, Cambridge University, Cambridge, United Kingdom; Mohammed Bin Rashid University of Medicine and Health Sciences, UNITED ARAB EMIRATES

## Abstract

Invasive Non-typhoidal *Salmonella* (iNTS) disease is a major public health challenge, especially in Sub-Saharan Africa (SSA). In Kenya, mortality rates are high (20–25%) unless prompt treatment is instituted. The most common serotypes are *Salmonella enterica* serotype Typhimurium (*S*. Typhimurium) and *Salmonella enterica* serotype Enteritidis (S. Enteritidis). In a 5 year case-control study in children residing in the Mukuru informal settlement in Nairobi, Kenya, a total of 4201 blood cultures from suspected iNTS cases and 6326 fecal samples from age-matched controls were studied. From the laboratory cultures we obtained a total of 133 *S*. Typhimurium isolates of which 83(62.4%) came from cases (53 blood and 30 fecal) and 50(37.6%) from controls (fecal). A total of 120 *S*. Enteritidis consisted of 70(58.3%) from cases (43 blood and 27 fecal) and 50(41.7%) from controls (fecal).

The *S*. Typhimurium population fell into two distinct ST19 lineages constituting 36.1%, as well as ST313 lineage I (27.8%) and ST313 lineage II (36.1%) isolates. The *S*. Enteritidis isolates fell into the global epidemic lineage (46.6%), the Central/Eastern African lineage (30.5%), a novel Kenyan-specific lineage (12.2%) and a phylogenetically outlier lineage (10.7%). Detailed phylogenetic analysis revealed a high level of relatedness between NTS from blood and stool originating from cases and controls, indicating a common source pool. Multidrug resistance was common throughout, with 8.5% of such isolates resistant to extended spectrum beta lactams. The high rate of asymptomatic carriage in the population is a concern for transmission to vulnerable individuals and this group could be targeted for vaccination if an iNTS vaccine becomes available.

## Author summary

Blood-stream infections in young children in Sub-Saharan Africa cause high levels of morbidity and mortality. Non-typhoidal *Salmonella* (NTS) serovars Typhimurium and Enteritidis are especially important in causing blood-stream infections in Kenya. In this case-control study we examined a total of 4201 children with potential blood-stream infections and compared their NTS genotypes with those found in fecal samples of 6326 asymptomatic age-matched controls. From a phylogenetic analysis, we observed a high rate of carriage in cases and controls of multidrug resistant *Salmonella* genotypes with similarity to those causing invasive disease. We hypothesize that the high carriage rates in asymptomatic population may be contributing to maintenance and transmission of NTS disease among vulnerable population of children.

## Introduction

Non-typhoidal *Salmonella* (NTS) disease is responsible for an estimated 3.4 million global cases annually, where it is predominantly associated with gastroenteritis. In most parts of the world NTS is not normally associated with invasive (bloodstream) disease. However, in Sub-Saharan Africa (SSA) there is a high burden of this type of disease, particularly in infants, with an untreated case fatality rate between 20 to 25% [1–5]. Among iNTS cases in SSA, it is estimated that ~65% occur in children <5 years of age [1], with a range from 166-568/100,000 cases of person-years of observation (pyo). iNTS disease in SSA is more prevalent amongst Human Immunodeficiency Virus (HIV)-infected individuals, infants, and young children with malaria, anaemia and malnutrition [4].

Increasing antimicrobial resistance in iNTS is of great global concern where empiric oral options for effective treatment of life-threatening invasive disease are being rapidly eroded. Multi-drug resistance (MDR) (resistance to first line drugs such as ampicillin, sulpha-trimethoprim and chloramphenicol) in iNTS has been reported in Kenya and Malawi [6–8], and in other parts of SSA [9–11]. MDR *Salmonella* serotype Typhimurium of a novel sequence type (ST) 313 is endemic in SSA countries including Kenya [8], Malawi [6], the DRC [12], Nigeria [13], Ghana [14], South Africa [15] and Mozambique [16]. In these settingsST313 frequently produces septicemia in the absence of gastroenteritis. Recently we reported on the emergence of MDR ST 313 also resistant to extended spectrum beta lactams (ESBL) (including ceftriaxone) encoded on a CTX-M-15 gene located on a 304kb plasmid. Ceftriaxone is among the last-line antimicrobials used to treat severe life-threatening bacterial infection in our setting. iNTS isolates with resistance to ceftriaxone have also been reported in other countries in SSA: the Democratic Republic of the Congo (DRC) [12, 17], Malawi [18, 19] and South Africa [20]. MDR *S*. Enteriditis ST11, has also emerged in Kenya [21, 22] and Malawi [7], while in Ghana we have recent reports of emergence of fluoroquinolone resistance strains [23]. However, ESBL producing strains have not been reported.

While zoonotic animal reservoirs of iNTS ST313 remain unclear, it is hypothesized that iNTS infections can be transmitted through human-to-human contacts [22, 24]. Household asymptomatic carriers are thought to be a major reservoir and source for spread of infection to vulnerable individuals. Nairobi, Kenya’s largest urban centre, has an estimated population of 4.5 million, a third of who live in informal settlements that lack proper sanitary and clean water facilities. The dense population and sub-optimal hygiene and sanitation contribute to the high burden of diseases due to enteric pathogens in these settings. Here, we report on MDR iNTS from patients treated at outpatient clinics that have a high degree of phylogenetic relatedness to strains isolated from asymptomatic carriers; indicating possible human-human transmission in the community settings.

## Materials and Methods

### Study site

**Fig 1:**
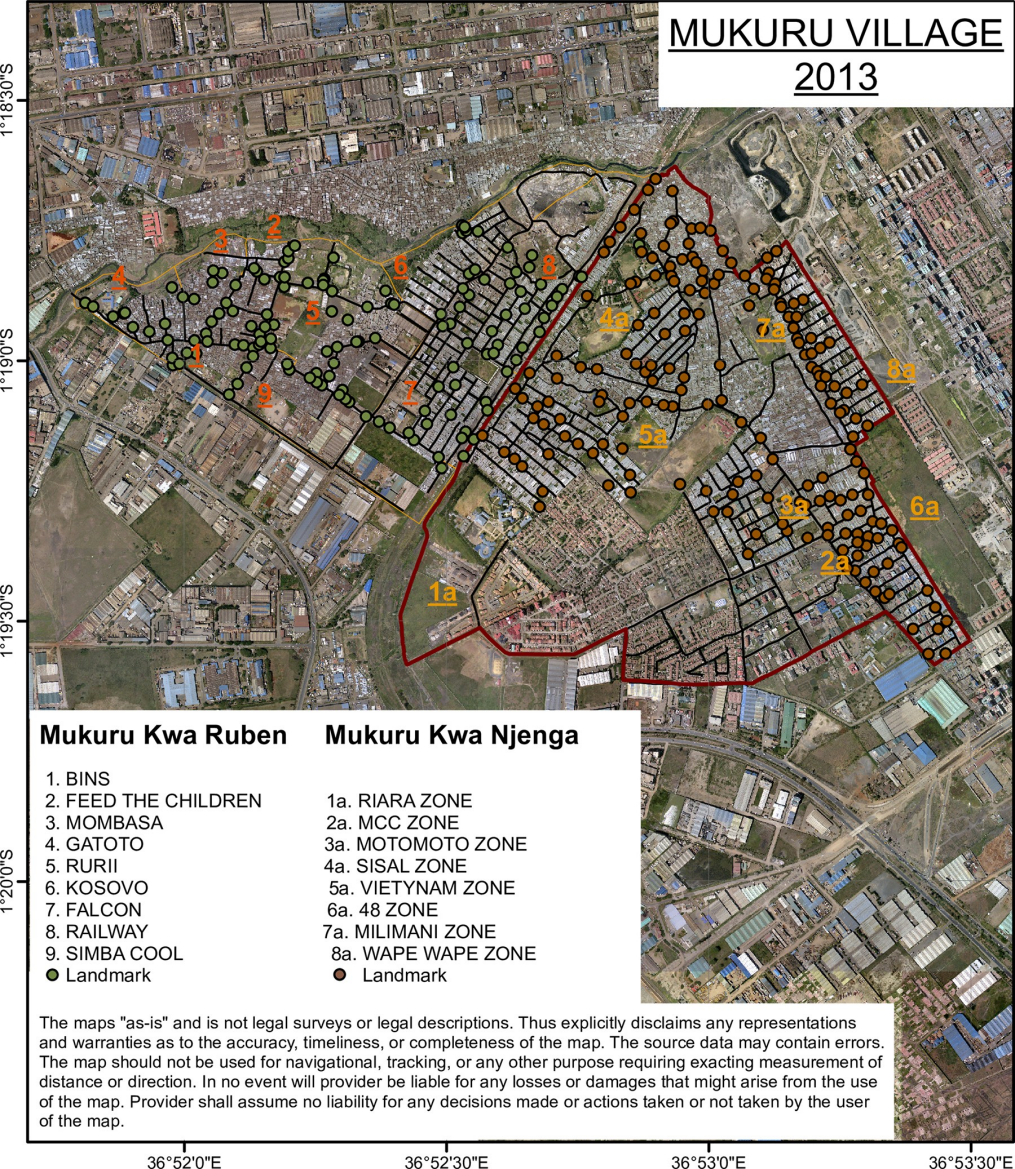
Mapped villages constituting Mukuru informal settlement study site. The image was acquired from Google Earth to create the map of structures/buildings of the study area as the first step of creating the GIS database. The images were geometrically rectified to a known coordinate system (Universal Transverse Mercator (UTM) 37S on the basis of a number of Ground Control Points (GCPs), selected from the periphery of the study area so that possible errors would converge towards middle of the area. Images were converted into TIFF files in ArcGIS10x software.

Mukuru informal settlement is situated in the eastern side of Nairobi about 15 KM from the city centre. It is one of the largest slums in the city with a population of around 250,000 people [25]. Informal settlements are made up of improvised temporary dwellings often made from scrap materials, such as corrugated metal sheets, plywood, and polythene-sheets. The informal settlements are densely populated and characterized by limited basic services and infrastructure for providing clean water, sanitation facilities, solid-waste management, roads, drainage, and electricity [26]. In addition to poverty, a number of factors associated informal settlements, including overcrowding, substandard housing, unclean and insufficient quantities of water, and inadequate sanitation, contribute to a high incidence of infectious diseases and increased mortality among those under five years [27, 28]. Mukuru Informal settlement is divided into eight villages; Mukuru Lunga-Lunga, Mukuru kwa Sinai, Mukuru kwa Reuben, Mukuru kwa Njenga, Mukuru Kayaba and Mukuru North. The two major villages within the larger Mukuru, Mukuru kwa Njenga and Mukuru kwa Reuben, were mapped. This was done through digitizing detailed physical structures, creation of geographic data of road networks and projection of geographic data in a plane using Universal Transverse Mercator system. Mukuru Reuben was demarcated into 9 zones with 7037 blocks, while Mukuru kwa Njenga had 8 zones with 8059 blocks (Fig 1). In total, 32,000 households were mapped.

### Study subjects and specimen collection

The study population residing in Mukuru informal settlment receive routine immunization for common diseases within the Expanded programme on immunization. Patients presenting for care at 4 outpatient medical facilities that serve the informal settlement were approached for participation in the study if they were <16 years of age on the date of presentation; resided in the Mukuru slum; presented with a subjective history of at least 3 days of fever and have an axillary temperature of at least 37.5 ^o^C or they presented with a history of fever of any duration and have an axillary temperature of at least 37.5°C; or they reported having had three or more loose or liquid stools (children > 2 years) or 8 or more for infants in the 24 hours before presentation, or one or more loose or liquid stool with visible blood. Eligible patients whose guardians gave written informed consent had a detailed history and physical examination recorded on a structured data form and a blood and stool specimen taken for culture. A structured questionnaire (S1 Table) was used to elucidate the following information from each case after blood and stool (or rectal swabs for those that stool samples were unavailable) samples were collected: clinical manifestations (e.g. vomiting, fever, and/or dehydration), demographic data (age, sex, and residence), and types of stool samples (watery, mucous, or bloody, or other form). Controls recruited for the study were age-matched children who came to the health facility for well mother and child clinic including routine vaccinations. Whenever available, two age-matched controls were randomly selected for each case identified. A questionnaire was used to obtain medical history of the control, and their demographic data (age, sex, and residence). From each control, a stool or rectal swab were obtained and recorded as formed, watery, mucous, bloody, or other form.

### Detection of bacterial pathogens

#### Blood for culture

For blood culture 1–3 ml for children < 5 years of age and 5–10 ml for 5–16 years of age was collected in syringe, placed into culture media in Bactec bottles, and transported daily to and analyzed at the KEMRI laboratory. Blood cultures were incubated at 37°C in a computerized BACTEC 9050 Blood Culture System (BD, Franklin Lakes, New Jersey, USA), and subcultured after 24 h onto blood, chocolate and MacConkey agar plates. The blood cultures were subsequently observed for a further 7 days for signs of bacterial growth (auto-detection). A final subculture was performed for all blood cultures on the 8th day regardless of the state of bacterial growth. From the subcultures, bacterial isolates were identified using biochemical tests on API20E strips (API System, Montalieu Vercieu, France) and further typed by species-specific serological tests.

#### HIV serology/PCR tests

Comprehensive testing for HIV for all children whose HIV status is not known is currently routine in each of the clinical study sites. After providing counselling by qualified HIV counsellors and informed consent from parents or guardians (and assent for children 7 years of age and older), a drop of blood was spotted onto Whatman absorbent filter paper (Schleicher & Schuell 903 or Whatman BFC 180). The samples were transported to KEMRI laboratory for testing. Children above 18 months of age were tested using the Determine HIV Rapid test and confirmed using the Uni-Gold Rapid HIV test kits. These are the kits approved by Ministry of Health. For children below 18 months of age, HIV status was evaluated by PCR. Any HIV seropositive children were referred to a clinic physician for further evaluation and management.

#### Malaria blood smear test

As malaria is a major differential diagnosis in children presenting with fever, a malaria smear (thick and thin) was made for all cases. The smear slides were Giemsa stained and read on-site. Results were entered into study cards and also given to the clinician for patient management.

#### Stool cultures

The rectal swab or loopful of the stool specimen was transported to KEMRI laboratory and initially cultured on selenite F (Oxoid, Basingstoke, UK) broth aerobically at 37°C overnight. Broth cultures were then subcultured on MacConkey agar and *Salmonella-Shigella* agar (Oxoid) and incubated at 37°C overnight. To identify suspect *Salmonella* bacteria, non-lactose fermenting colonies were biochemically tested using triple sugar iron (TSI) slants. From the subcultures, bacterial isolates were identified using biochemical tests on API20E strips and further typed by species-specific serological tests.

#### Antibiotic susceptibility testing

Antibiotic susceptibility testing was performed using the disk diffusion technique for all commonly used antimicrobials in Kenya on Mueller-Hinton agar (Oxoid, Basingstoke, UK). For Gram negative enteric bacterial spp this includes ampicillin 10μg, tetracycline 30μg, gentamicin 10μg, trimethoprim 5μg, sulphamethoxazole 100μg, chloramphenicol 30μg, co-amoxiclav 20:10μg, cefuroxime 30μg, ceftazidime 30μg, ceftriaxone 30μg, cefotaxime 30μg, ciprofloxacin 5μg and nalidixic acid 10μg. MICs were performed using the E-test strips (AB BIODISK, Solna, Sweden). Results were interpreted according to the guidelines provided by the Clinical and Laboratory Standards Institute, 2017 [29].

#### Genomic analysis of NTS isolates

Genomic DNA from the NTS isolates listed in S2 Table was extracted using the Wizard Genomic DNA Extraction Kit (Promega, Wisconsin, USA). Two μg of genomic DNA was subjected to whole genome sequencing (WGS) using Illumina sequencing for high resolution and high throughput sample analysis. Sequence data is made publicly available and accession numbers are included in S2 Table. Quality was checked through assembly and mapping statistics, and Kraken output was used to check for contamination. Genomics MLST was determined using the SRST2 software. *S*. Typhimurium sequences were mapped to *S*. Typhimurium D23580 (Accession Number = NC_016854), *S*. Enteritidis sequences to *S*. Enteritidis P125109 (Accession Number = NC_011294.1) using SMALT v0.7.4. Variation detection was performed using samtools mpileup v0.1.19 with parameters “-d 1000 -DSugBf” and bcftools v0.1.19 [30]. A pseudo-genome was constructed by substituting the base call at each site (variant and non-variant) in the BCF file into the reference genome and any site called as uncertain was substituted with an N. Insertions with respect to the reference genome were ignored and deletions with respect to the reference genome were filled with N’s in the pseudo-genome to keep it aligned and the same length as the reference genome used for read mapping. Plasmids and recombinant regions (prophages and CRISPR sequences) in the chromosome were removed from the alignment. SNP sites were extracted from the alignment using snp-sites [31] and used to construct a maximum likelihood phylogeny. RAxML v8.2.8 [32] with substitution model GTRCAT. Support for nodes on the trees was assessed using 100 bootstrap replicates. The S. Typhimurium strains and S. Enteritidis stains were aligned to reference genomes *Salmonella* Typhimurium D23580 and S. Enteritidis P125109, respectively, using a pipeline developed in-house at the Wellcome Trust Sanger Institute (WTSI). Trees were visualized using iTOL [33]. To identify the lineages, the sequences from this study were phylogenetically analysed within the African context of Feasey *et al*. and Van Puyvelde *et al*., [7, 34]. Resistance genes were determined from the raw Illumina sequencing data using ariba v 2.12.1 [35] with CARD database version 3.0.2 [36].

#### Ethical Considerations

The study was approved by the Scientific and Ethics Review Unit (SERU) of KEMRI (SSC No. 2076). All parents and/or guardians of participating children were informed of the study objectives and voluntary written consent was sought and obtained before inclusion. A copy of the signed consent was filed and stored in password protected cabinets at KEMRI.

#### Hypothesis

The hypothesis was that high carriage rates of NTS strains in non-symptomatic individuals in the community may facilitate human-to-human NTS transmission.

## Results

### Patients and controls

From the 4 health facilities, a total of 10,527 children were recruited into the study; 4,201 were cases and 6,326 were healthy controls. In the study, a total of 4201 blood cultures from suspect iNTS cases and stool samples from 6326 age-matched controls were obtained from children less than 5 years of age. The majority of the suspect iNTS cases (26.1%) were aged 13–24 months, with the number decreasing to 14.1% in the age group 49–60 months Table 1). 1.3% of the children were reported to be HIV positive, 4.1% tested malaria positive.

**Table 1:** Age distribution of patients, both cases and controls.

	Case	Control
Age category	No. (%)	No. (%)
0–12 months	811 (19.3)	846 (13.4)
13–24 months	1096 (26.1)	1812 (28.6)
25–36 months	875 (20.8)	1412 (22.3)
37–48 months	828 (19.7)	1304 (20.6)
49–60 months	591 (14.1)	952 (15.1)
**Total**	**4201 (100)**	**6326 (100)**

### Highly related case and control isolates form a diverse NTS population

**Fig 2:**
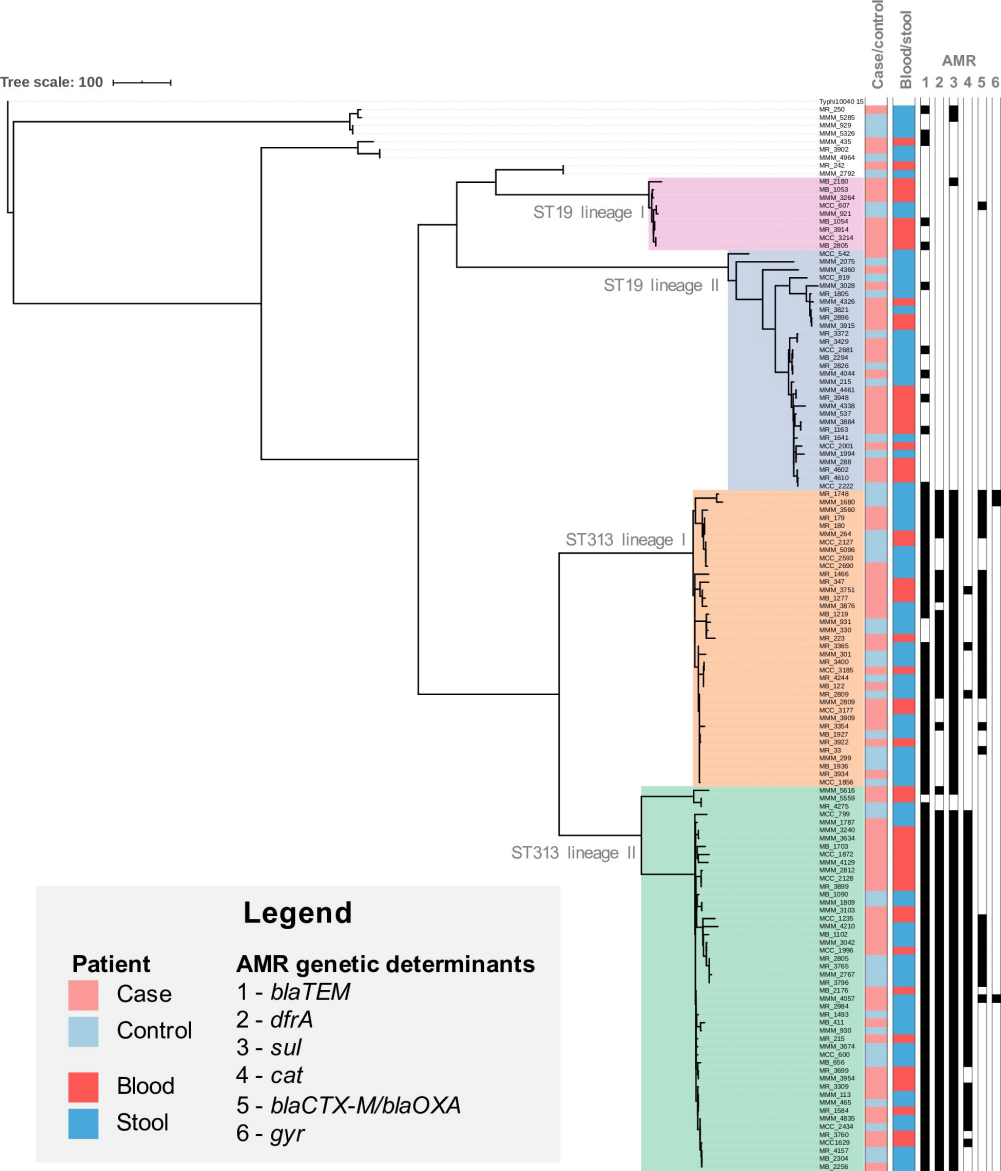
Phylogenetic tree of *S*. Typhimurium isolates from this study. Maximum likelihood phylogenetic tree based on the 130 *S*. Typhimurium genome sequences from this study. Sequencing reads were mapped to *S*. Typhimurium ST313 lineage II reference strain D23580, and *S*. Typhi 10040_15 was added as an outgroup to root the tree. The tree is based on 61161 chromosomal SNPs. Patient state (case/control) and the source (blood/stool) is visualized as indicated in the legend. Presence of multidrug resistance markers (MDR; *bla*, *dfrA*, *sul*, *cat*), *blaCTX-M/blaOXA* antimicrobial resistance markers and SNPs in *gyr* are annotated. Branch lengths represent numbers of SNPs as indicated in the scale bar.

**Fig 3:**
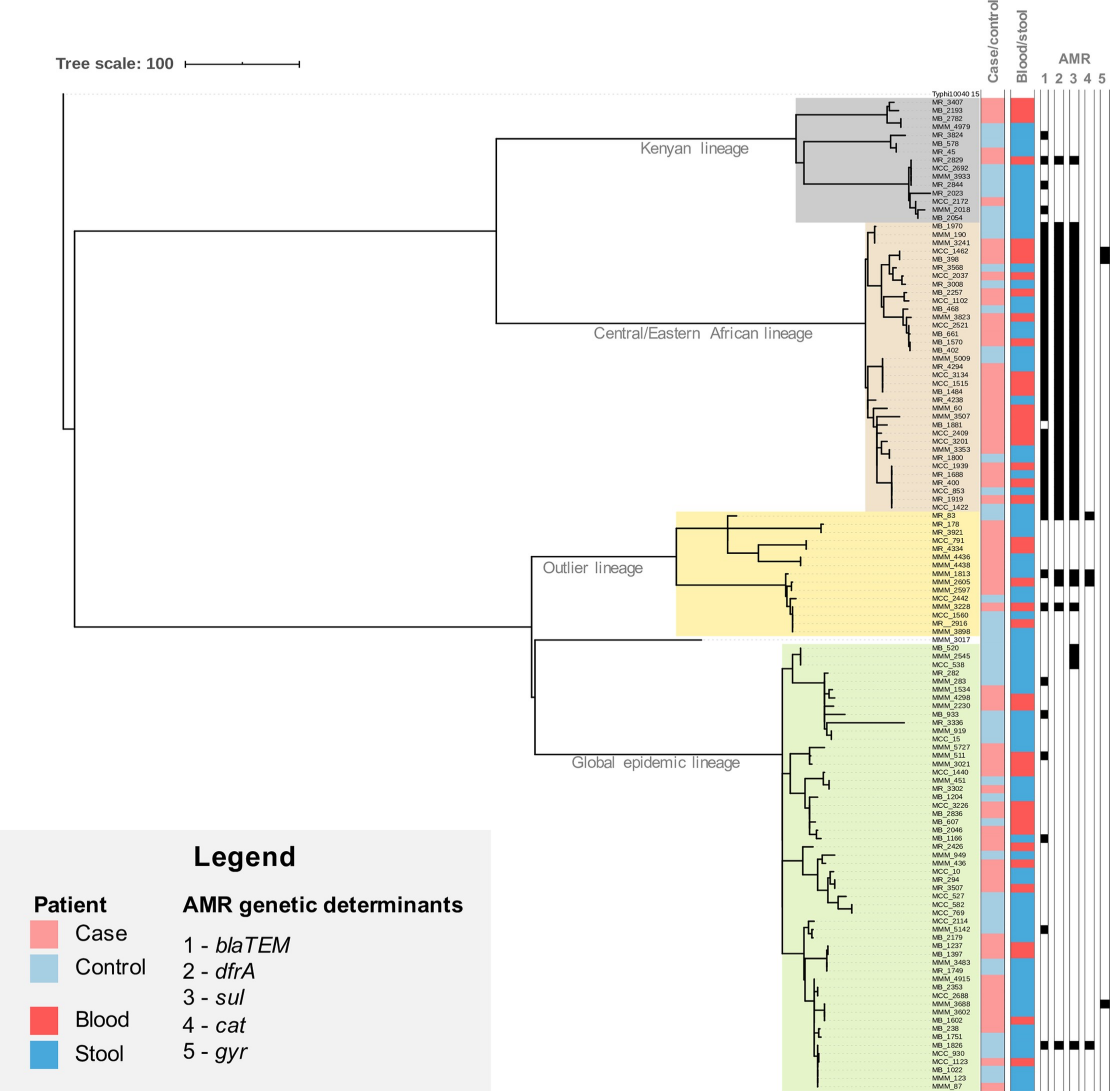
Phylogenetic tree of *S*. Enteritidis isolates from this study. Maximum likelihood phylogenetic tree based on the 120 *S*. Enteritidis genome sequences from this study. Sequencing reads were mapped against *S*. Enteritidis reference strain P125109, and *S*. Typhi 10040_15 was added as an outgroup to root the tree. The tree is based on 60302 chromosomal SNPs. Patient state (case/control) and the source (blood/stool) is visualized as indicated in the legend. Presence of multidrug resistance markers (MDR; *bla*, *dfrA*, *sul*, *cat*) and SNPs in *gyr* are annotated. Branch lengths represent numbers of SNPs as indicated in the scale bar.

From the 4201 blood cultures from cases and 6326 fecal samples from age-matched controls we obtained a total of 133 *S*. Typhimurium isolates of which 83(62.4%) came from cases (53 blood and 30 fecal) and 50(37.6%) from controls (fecal). A total of 120 *S*. Enteritidis consisted of 70(58.3%) from cases (43 blood and 27 fecal) and 50(41.7%) from controls (fecal). Whole genome sequencing and phylogenetic analysis showed that both the invasive and enteric *S*. Typhimurium (133 isolates) and *S*. Enteritidis (120 isolates) form two distinct bacterial populations with different substructures (Figs 2 and 3), but with minimal phylogenetic differences between invasive and enteric isolates. The *S*. Typhimurium isolates harbour 4389 and the *S*. Enteritidis isolates 3175 core SNPs difference.

Whereas the current pandemic of invasive *S*. Typhimurium isolates across SSA is dominated by ST313 lineage II isolates, the Kenyan *S*. Typhimurium population is more diverse and falls into four lineages; two ST19 lineages (36.1%), ST313 lineage I (27.8%) and ST313 lineage II (36.1%) (Fig 2 and S1 Fig).

The *S*. Enteritidis isolates fell into four different lineages; the global epidemic lineage (46.6%), a so called outlier lineage (10.7%), a Central/Eastern African lineage (30.5%) as defined by Feasey *et al*. (2016) [7], and a novel Kenyan lineage (12.2%) (Fig 3 and S2 Fig).

In all *S*. Typhimurium and *S*. Enteritidis lineages, the isolates from blood and stool, originating from cases and controls were randomly distributed across the phylogenetic tree. In addition, in 8 cases the serotypes isolated from blood and from stool samples from the same individual were different (*S*. Enteritidis and *S*. Typhi, 4; *S*. Typhimurium and *S*. Typhi, 2; *S*. Typhimurium and *S*. Enteritidis, 2). However, corresponding isolates from individual cases and controls showed high genetic relatedness, with some examples where identical genotypes (0 core genome SNPs difference) were found among cases and controls.

### NTS lineages have heterogeneous distributions of antimicrobial resistance genes

A total of 150/253(59.3%) NTS were resistant to 2 or more commonly available antimicrobials. The most common resistance phenotype were resistance to ampicillin, chloramphenicol, and sulphamethoxazole-trimethoprim (70%). In addition, 23(9.1%) isolates were resistant to extended spectrum beta lactams (cefotaxime or ceftriaxone or ceftazidime), and one isolate was resistant to ciprofloxacin. Using the Yates chi-square test there was no significant relationship between MDR phenotype with either age, co-morbidity with malaria, sickle cell trait or HIV status.

The presence of AMR genetic markers was correlated with specific phylogenetic clades, irrespective of the source of the isolate (case or control). Isolates that fell in *S*. Typhimurium ST313 lineages I and II and the Central/Eastern *S*. Enteritidis lineage show the highest AMR levels, with high abundance of MDR resistance genes and presence of ESBL genes (Figs 2 and 3). The majority of isolates that fell in *S*. Typhimurium ST19 lineages I and II (30/39; 77.0%) and the *S*. Enteritidis global epidemic lineage (44/54; 81.5%), did not harbor common genetic markers for AMR. Resistance genes contributing to the MDR phenotype (*blaTEM*, *dfrA*, *sul* and *cat*) were present in 52(39%) *S*. Typhimurium isolates and 3(2.5%) *S*. Enteritidis isolates. A total of 36(27.1%) *S*. Typhimurium carried ESBL genes (*blaCTX-M* and *blaOXA-1*). SNPs associated with decreased susceptibility to ciprofloxacin was identified in 3 *S*. Typhimurium isolates (GyrA D87Y, n = 2 and GyrB S463A, n = 1) and 3 *S*. Enteritidis isolates (*Gyr*A D87Y, n = 2 and *Gyr*A T83I, n = 1). Of note, one *S*. Typhimurium isolate showed resistance markers for MDR, ESBL activity and a ciprofloxacin resistance marker, the hallmarks of an extensively drug resistant (XDR) strain.

### Prevalence of HIV and malaria in the study population

The prevalence of HIV was 2.4% among cases and 3.8% among controls, while that of malaria was 4.1% among the cases and 1.8% among controls. Analysis for correlation of HIV and malaria prevalence with presence of iNTS disease did not show any significant association.

## Discussion

Invasive NTS disease in SSA continues to be a major cause of childhood morbidity and mortality [4, 6, 12, 22]. In Kenya the incidence ranges from 166-625/100,000 pyo, with mortality up to 28% [22]. In our settings, we observed a prevalence of 4.3%, very similar to rates observed in Malawi [6], Burkina Faso [37] and the DRC [12]. The Mukuru informal settlement is densely populated, and with poor WaSH characteristics, which facilitate the transmission of enteric infections, including iNTS strains, in this endemic setting.

The two main invasive *S*. *enterica* serotypes were *S*. Typhimurium (52.5%) and *S*. Enteritidis (47.5%). Similar patterns of serotypes causing iNTS disease in SSA have been described previously in Malawi [6], Ghana [14], the DRC [17, 38] Mozambique [39] and Burkina Faso [37]. From the analysis of genomic data, the population of *S*. Typhimurium is moderately diverse, with ST313 lineage I and ST313 lineage II being most common (63.9%), and ST19 lineages at 36.1%. In addition in 8 cases the serotypes isolated from blood and from stool samples of children were different (*S*. Enteritidis and *S*. Typhi, 4; *S*. Typhimurium and *S*. Typhi, 2; *S*. Typhimurium and *S*. Enteritidis, 2). This clearly indicates the unique multiplicity of *Salmonella* serotypes causing invasive disease in this population. Other studies in SSA [37, 39, 40] also observed similar proportions for ST313 lineages I and II, but fewer ST19 strains. In Kenya it appears that ST19 is also an important cause of iNTS disease as well asymptomatic carriage. ST19 has also been described in other studies on iNTS SSA but at lower prevalence rates [4, 16, 41].

The population structure of *S*. Enteritidis was also diverse, with the most common lineages (The East and Central Africa and the Global lineages), also being commonly found in other SSA iNTS endemic countries [37, 42]. However, we observed a novel Kenyan lineage *S*. Enteriditis (12.2%). The diverse population structure of both *S*. Typhimurium and *S*. Enteritidis that form the main iNTS strains causing disease in Kenya could have implications for vaccine development and coverage for candidate vaccines currently under development.

In this case-control study, analysis of proportions by the Yates chi-square test showed a high correlation (p-value <0.01) between *S*. Typhimurium and *S*. Enteritidis genotypes in cases and controls. This implies a potentially important role for asymptomatic carriage in the community and this silent carriage could serve as possible source of infection for vulnerable individuals. In addition, these findings further indicate a possible role for human-human transmission as an important route for spread of iNTS disease in these endemic settings [24]. It will be crucial to understand the dynamics of transmission in these settings so we can target the asymptomatic carriers for possible vaccination.

The *S*. Typhimurium and *S*. Enteritidis lineages previously linked to iNTS disease in Africa including ST313 lineages I and II, and the Central/Eastern African Enteritidis lineage [42] show the highest rates of antimicrobial resistance. In agreement with these previous observations, isolates from this study that fall in *S*. Typhimurium ST313 lineages I and II and the Central/Eastern *S*. Enteritidis lineage show the highest antimicrobial resistance levels, with high abundance of MDR genes and presence of ESBL genes. The most common resistance genes associated with MDR were *blaTEM*, *dfrA*, *sul* and *cat*. These MDR NTS lineages have previously been described for their importance in the SSA iNTS pandemic [40, 42]. These MDR genes on plasmids are self-transferrable between species and may play a major role in the spread of AMR in the enteric bacteria population. One isolate had co-resistance of ciprofloxacin and ceftriaxone; these drugs are treatment options of last resort for severe iNTS disease. These isolates bear the characteristics of Extremely Drug Resistant (XDR) phenotype as previously described [34, 41, 43].

In conclusion, we observed a high prevalence of NTS carriage in the study population, which may provide a significant reservoir for transmission, and maintenance of invasive disease in the population. Multidrug resistance including increasing resistance to extended spectrum beta-lactams and reduced susceptibility to fluoroquinolones presents a huge challenge for management of severe infections in this population. As we face the challenges of treating MDR iNTS disease, it is important to note that alternative effective options for management of iNTS disease are dwindling. Indeed other options are often unavailable or too expensive to be affordable to patients attending the public healthcare facilities. Where new effective antimicrobials are lacking, developments in vaccines may offer hope for reducing the burden of iNTS in endemic settings in SSA. A bivalent efficacious vaccine, targeting *Salmonella* serovars Typhimurium and Enteritidis, would significantly lower the disease burden in high-risk populations especially as asymptomatic carriage in the endemic settings is high.

## Supporting information

S1 Table Clinical examination data form. (DOCX)

S2 Table List and Accession numbers for non-typhoidal *Salmonella* (NTS) isolates used in this study. (XLS)

S1 Fig Phylogenetic Tree of *S*. Typhimurium showing the single nucleotide polymorphism (SNP) differences between lineages. (JPG)

S2 Fig Phylogenetic Tree of *S*. Enteritidis showing the single nucleotide polymorphism (SNP) differences between lineages. (JPG)

S3 Fig *S*. Typhimurium lineages and their genetic relatedness in context. (JPG)

S4 Fig *S*. Enteritidis lineages and their genetic relatedness in context. (JPG)
