# Supplementary material for: High relatedness of invasive multi-drug resistant non-typhoidal Salmonella genotypes among patients and asymptomatic carriers in endemic informal settlements in Kenya
Source: PLoS Negl Trop Dis. 2020 Aug 3;14(8):e0008440. doi: 10.1371/journal.pntd.0008440 (PMC7425985; doi:10.1371/journal.pntd.0008440)
Supplement: S1 Table — (DOCX) [file pntd.0008440.s001.docx]

## IDENTIFYING INFORMATION

| Please affix a label here.   1. **Personal ID number** 2. **Date of visit** 3. **Name of patient** 4. **Sex** 5. **Date of birth** 6. **Age** |
| --- |

If date of birth was not given in question 5 in the label, complete

Date of birth: __ __/ __ __/ __ __ __ __

D D M M Y Y Y Y

**[I] CLINICAL SYMPTOMS**

1. Did you have fever in past 3 days? Yes No Not sure

a) If yes, was it continuous fever? Yes No Not applicable

b) If yes, for how many days? _______________ (days)

2. Does the subject have any past or current symptom of the following in past 4 weeks?

|  |  | Yes | No | DK |  | Yes | No | DK |
| --- | --- | --- | --- | --- | --- | --- | --- | --- |
| **Respiratory** | Cough |  |  |  | Expectoration |  |  |  |
|  | Hemoptysis |  |  |  |  |  |  |  |
| **G-I** | Vomiting |  |  |  | Diarrhea |  |  |  |
|  | Abdominal Pain |  |  |  | Blood diarrhea |  |  |  |
|  | Distension |  |  |  | Constipation |  |  |  |
| **G-U** | Dysuria |  |  |  | Passing urine  more frequently then usual |  |  |  |
| **Neurology** | Headache |  |  |  | Seizure |  |  |  |

## [II] PAST ILLNESS HISTORY

4. Does the subject have any past or current disorder of the following?

|  | Yes | No | Tick box  If present |  | Yes | No | Tick box  If present |
| --- | --- | --- | --- | --- | --- | --- | --- |
| Sickle cell disease |  |  |  | Malignancy |  |  |  |
| Tuberculosis |  |  |  | HIV/AIDS |  |  |  |
| Sickle cell disease |  |  |  | Typhoid Fever |  |  |  |

## [III] PAST MEDICATION HISTORY

5. Did you take any medication in the last 8 weeks? Yes No Not sure

→If yes, which medication did you take?

| Chloramphenicol Amoxicillin Co-Amoxiclav Ciprofloxacin Ceftriaxone  Anti-malarial drugs Others, specify_______________ Not applicable  Don’t know |
| --- |

## [IV] VITAL SIGNS

6. Measure items below and write the results.

| Heart Rate | \|  \|  \|  \| \| --- \| --- \| --- \|   (beats per minute) | Information not obtained |
| --- | --- | --- | --- | --- | --- |
| Temperature | \|  \|  \| **.** \| // \| \| --- \| --- \| --- \| --- \|   °C  Oral Rectal Axillary Other | Information not obtained |
| Respiratory Rate | \|  \|  \| \| --- \| --- \|   (Breaths per minute) | Information not obtained |

## [V] PHYSICAL MEASUREMENTS

7. Measure items below and write the results.

| Height | \|  \|  \|  \| **.** \| // \| \| --- \| --- \| --- \| --- \| --- \|   (cm) | Information not obtained |
| --- | --- | --- | --- | --- | --- | --- | --- |
| Weight | \|  \|  \|  \| **.** \| // \| \| --- \| --- \| --- \| --- \| --- \|   (Kg) | Information not obtained |
| Mid-arm circumference | \|  \|  \|  \| **.** \| // \| \| --- \| --- \| --- \| --- \| --- \|   (cm) | Information not obtained |

## [VI] PHYSICAL EXAMINATION

8. After physical examination, tick relevant box.

| General | Cyanosis edema |
| --- | --- |
|  | Dehydration: None mild moderate severe |
| HEENT | Coated tongue Jaundice Pallor(conjunctiva) |
|  | Throat: Normal Pharyngotonsillitis Others |
| CNS | Consciousness level:  Normal apathy disorientation delirium stupor |
|  | Neck stiffness Focal Neurological signs |
| Abdomen | Distension tenderness Palpable liver Palpable spleen  Decreased abdominal sound Rebound tenderness |
| Chest | Chest indrawing Bronchial Breath Rhonchi Crepitation |
| Others | Rose spot |

## [VII] Diagnosis & Treatment

9. Indicate lab diagnosis by blood culture and other tests? Typhoid Other Salmonella (NTS) spp  Other bacteria (specify to follow up) Not sure

a) If preliminary diagnosis was not typhoid fever or NTS, specify. _____________________________

10. Patient disposition? Sent home Admitted Died Absconded /loss Transferred to another project health care facility

Transferred else where ________________________

11. Was therapy initiated? Yes No Not applicable

a) If yes, which medication did you take (multiple choices are allowed)?

| Chloramphenicol Amoxicillin Co-amoxiclav Ciprofloxacin Ceftriaxone  antimalarial drugs others, specify_______________ Not applicable I don’t know |
| --- |

#

## [VIII] LABORATORY INVESTIGATIONS REQUEST

|  | Yes | No | NA |  |
| --- | --- | --- | --- | --- |
| Blood Sample taken |  |  |  | Lab Ref. Number  **please stick lab number label here, the same in tube & laboratory result form** |
| Stool/rectal Sample taken |  |  |  | Lab Ref. Number  **please stick lab number label here, the same in tube & laboratory result form** |
| Blood culture requested |  |  |  |  |
| Stool/swab culture requested |  |  |  |  |
| Whole blood count requested |  |  |  |  |
| HIV test consented/ requested |  |  |  |  |
| Widal test requested |  |  |  |  |
| Malaria film requested |  |  |  |  |
| Sickle cell test requested |  |  |  |  |

|  | Code/Initial | Signature | Date  (DD/MM/YYYY) |
| --- | --- | --- | --- |
| Patient examined & assessed by |  |  | __ __/__ __/__ __ __ __ |
| Form collected & revised by |  |  | __ __/__ __/__ __ __ __ |
| First data entry |  |  | __ __/__ __/__ __ __ __ |
| Second data entry |  |  | __ __/__ __/__ __ __ __ |
