# Supplementary figures and images for: High relatedness of invasive multi-drug resistant non-typhoidal Salmonella genotypes among patients and asymptomatic carriers in endemic informal settlements in Kenya
Source: PLoS Negl Trop Dis. 2020 Aug 3;14(8):e0008440. doi: 10.1371/journal.pntd.0008440 (PMC7425985; doi:10.1371/journal.pntd.0008440)

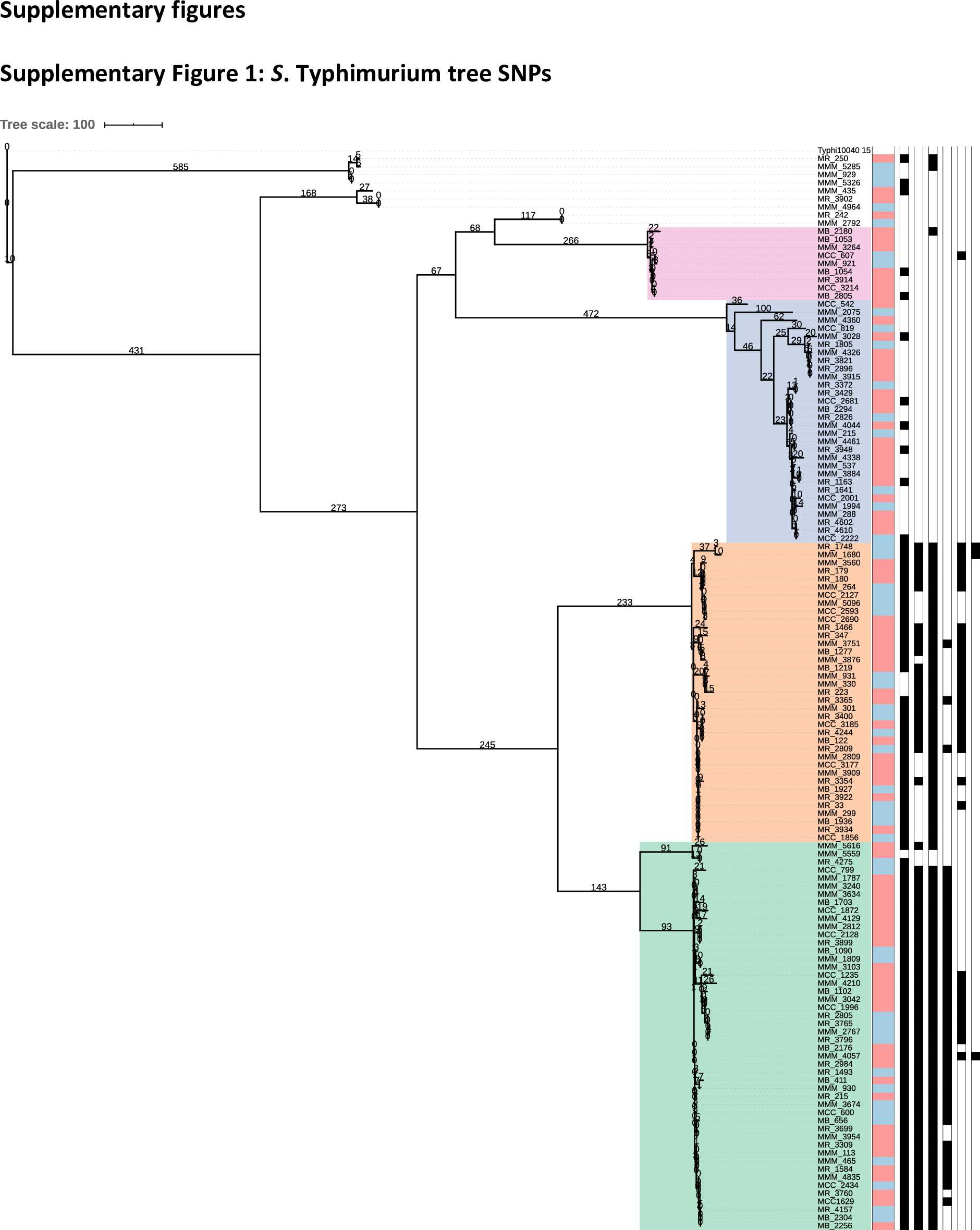

Supplement: S1 Fig — (JPG) [file pntd.0008440.s003.jpg]

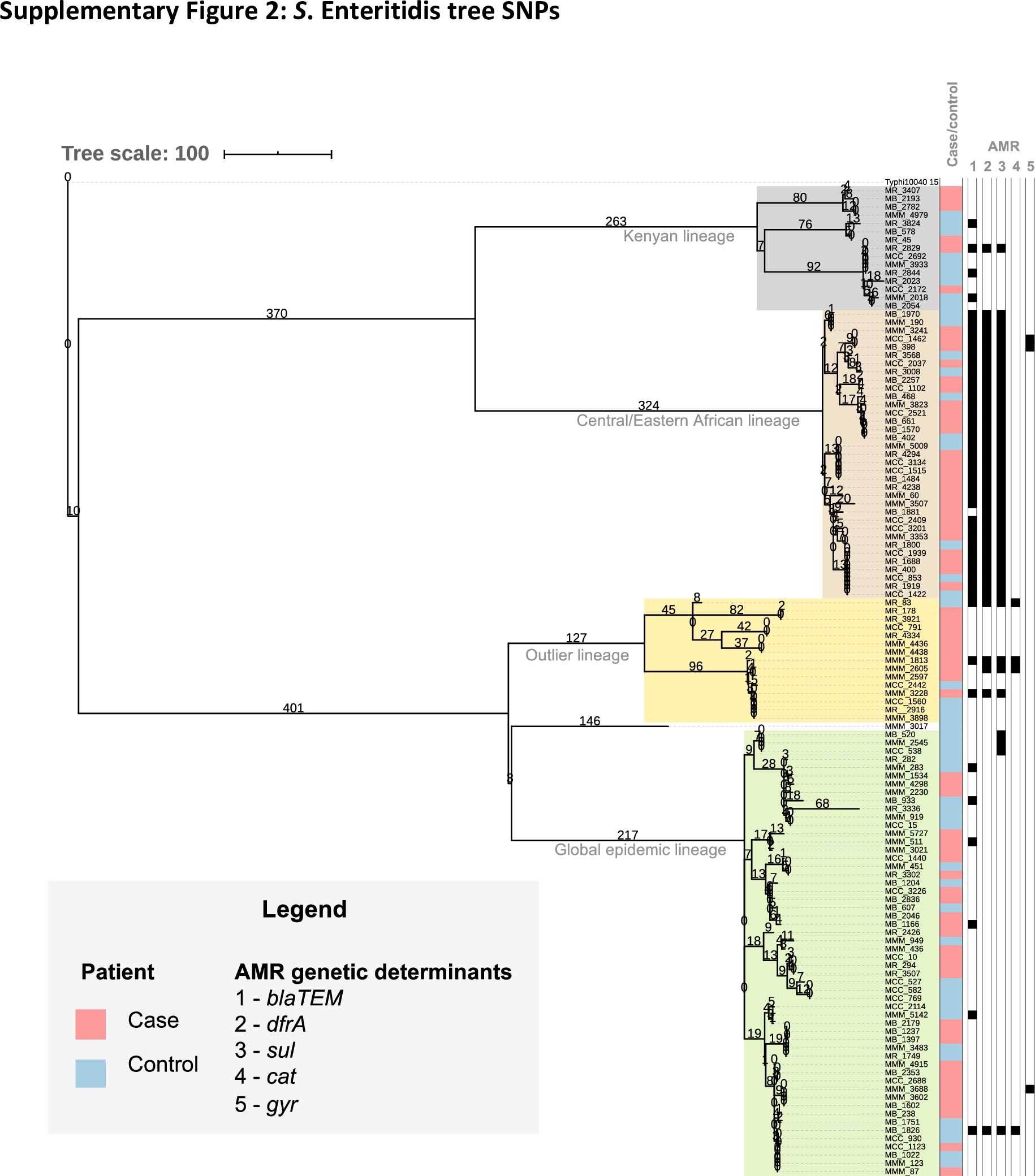

Supplement: S2 Fig — (JPG) [file pntd.0008440.s004.jpg]

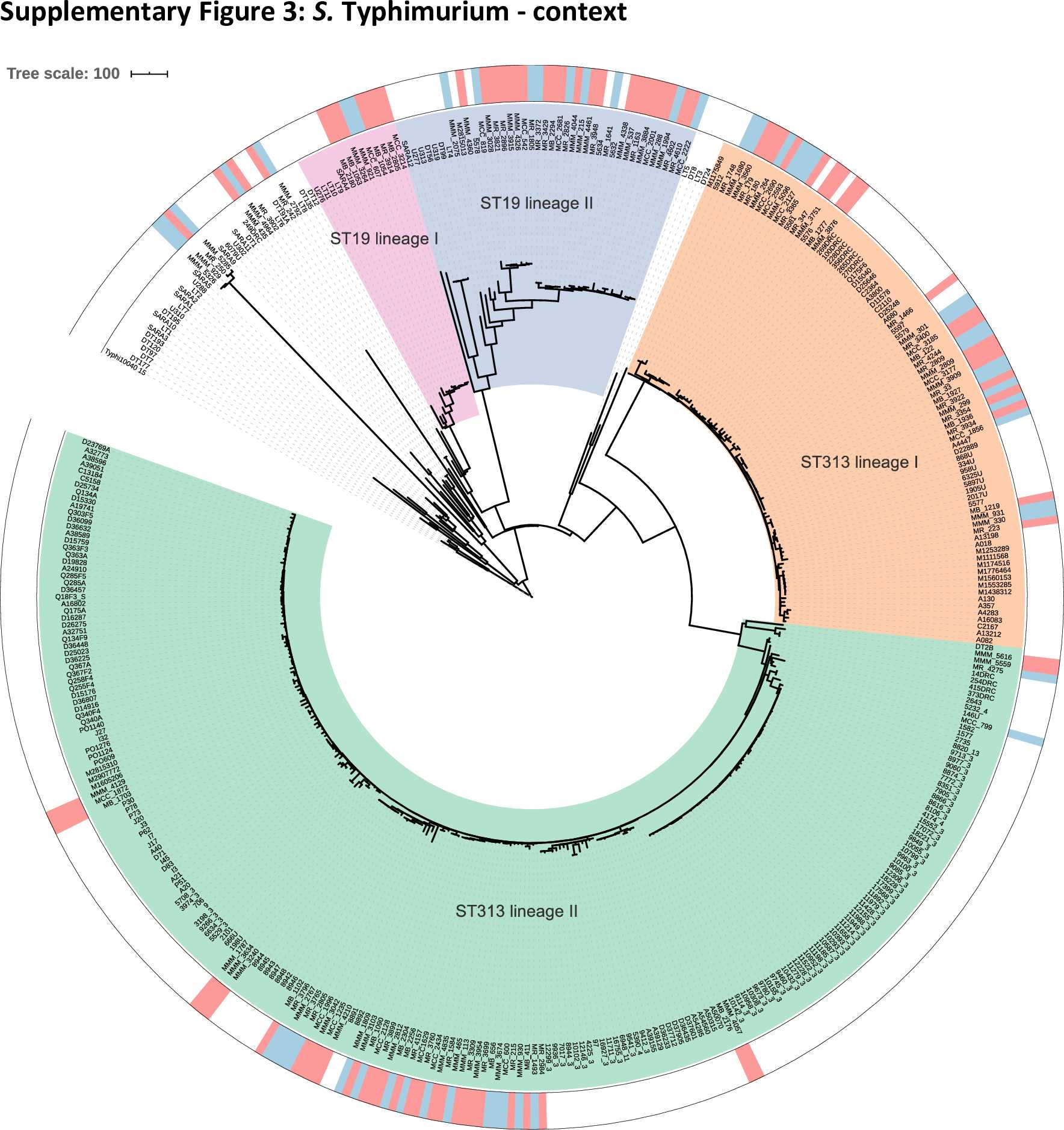

Supplement: S3 Fig — (JPG) [file pntd.0008440.s005.jpg]

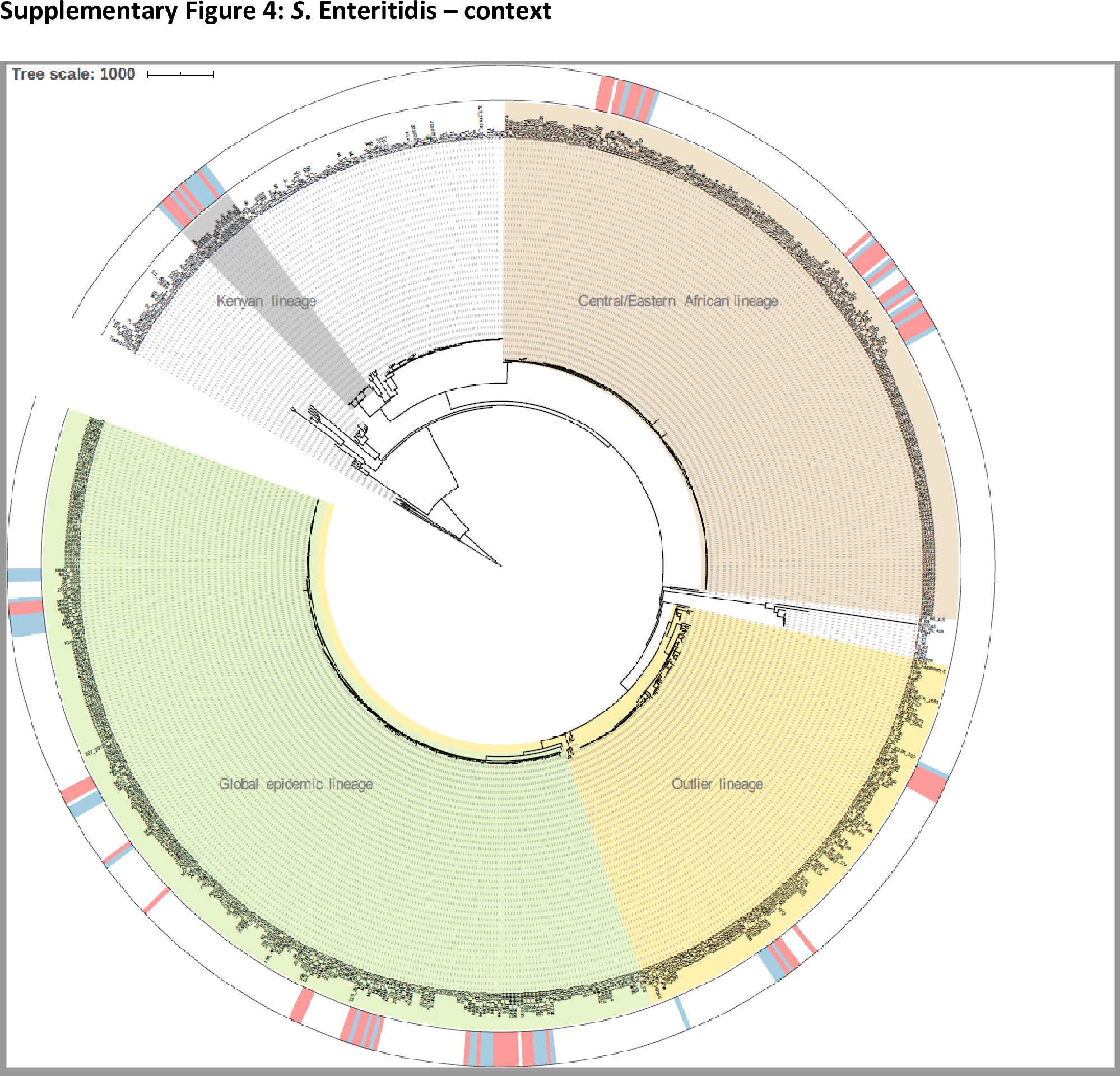

Supplement: S4 Fig — (JPG) [file pntd.0008440.s006.jpg]
